# Sexual dimorphism-driven differences are overcome in a preclinical vaccine model against *Trypanosoma cruzi*

**DOI:** 10.3389/fimmu.2025.1526573

**Published:** 2025-06-26

**Authors:** Camila Bulfoni Balbi, Maria Florencia Pacini, Brenda Dinatale, Cecilia Farré, Paula Cacik, Estefanía Prochetto, Florencia Belén González, Iván Marcipar, Gabriel Cabrera, Ana Rosa Pérez

**Affiliations:** ^1^ Instituto de Inmunología Clínica y Experimental de Rosario (IDICER-CONICET), Facultad de Ciencias Médicas, Universidad Nacional de Rosario, Rosario, Argentina; ^2^ Centro de Investigación y Producción de Reactivos Biológicos (CIPReB), Facultad de Ciencias Médicas, Universidad Nacional de Rosario, Rosario, Argentina; ^3^ Laboratorio de Tecnología Inmunológica, Facultad de Bioquímica y Ciencias Biológicas, Universidad Nacional del Litoral, Santa Fe, Argentina

**Keywords:** sexual dimorphism, Chagas disease, mucosal vaccines, trans-sialidase, MDSCs, regulatory T-cells, myocarditis, testosterone

## Abstract

Currently, no vaccine is available to prevent Chagas disease. Experimental vaccines against *Trypanosoma cruzi* (*Tc*) have shown high protection, but their development for humans still requires further study. Additionally, the sexual dimorphism observed in Chagas disease, with greater resistance in women, highlights the need to include both sexes in vaccine research to avoid biases. To assess the impact of sex on a recombinant vaccine, its immunogenicity and efficacy after oral infection in male and female BALB/c mice were evaluated. Additionally, gonadectomized (Gx) and sham-operated (Ms) males were used to estimate testosterone’s effect. The vaccine consisted of a recombinant fragment of *Tc*-derived *trans*-sialidase (TS) formulated with a cyclic-di-adenylate known as c-di-AMP (A), administered intranasally in three doses, 2 weeks apart. Control groups received TS alone, A, or a vehicle. Immunogenicity results showed that sexual dimorphism persisted after TS+A vaccination, with females having higher TS-specific IgG_2a_, IgG_1_, IgA, IL-17, and IFN-γ levels, while males showed greater delayed-type hypersensitivity and increased TS-specific IFN-γ^+^ROR-γt^+^ T-cell proliferation. Gx-TS+A-vaccinated males showed enhanced TS-specific IgG but not IgA, with negative effects on T-cell proliferation and higher parasite loads. Notably, after oral challenge with *Tc*, both sexes vaccinated with TS+A controlled parasitemia, reduced tissue parasite load, improved clinical outcomes, and attenuated myocarditis. In males, the vaccine also prevented the parasite-induced increase in splenic myeloid-derived suppressor cells (MDSCs) and preserved CD4^+^FoxP3^+^ regulatory T cells. Overall, TS+A nasal vaccination enhanced protection in both sexes, overcoming sexual dimorphism and highlighting its potential for human vaccine development.

## Introduction

Chagas disease (ChD) is caused by *Trypanosoma cruzi* (*Tc*). This illness affects nearly 6–7 million people, causing 12,000 deaths annually, mainly by cardiac causes. Treatment options include drugs having limited effectiveness during the chronic phase and often causing severe side effects, leading to potential treatment cessation. Presently, preventive strategies emphasize vector control, blood screening, and prenatal care since there is no vaccine available for ChD (1).

Numerous proof-of-concept studies on vaccines for ChD have been published with different antigens and delivery systems (2, 3). In many cases, protection is high, either by enhancing survival against parasite challenges or by preventing tissue damage after infection. This suggests that the development of vaccines for humans could be possible. However, many aspects still need to be evaluated in order to move in that direction. In experimental ChD, a clear sexual dimorphism is evidenced, with females demonstrating greater resistance than males (4). Furthermore, it was reported that in humans, *Tc*-infected male patients were at higher risk of infection (by enhanced exposition to vectors and/or outdoor activities) and myocarditis progression (5, 6). Despite that, experimental vaccine research often focuses predominantly on women, leading to potential biases in the results. Consequently, including both sexes in studies on vaccine responses is becoming increasingly important (7).

Indeed, sexual dimorphism may be driven by both intrinsic and extrinsic factors potentially impacting immunogenicity and protective efficacy. Among extrinsic regulatory factors, sexual hormones can either diminish or enhance the immune response, modulating the immunogenicity and immunocompetence (8). Estrogen enhances antibody production in women, which partly explains why women often handle infections more effectively and show a stronger humoral response to vaccinations compared to men. On the contrary, testosterone has clear suppressive effects on the humoral response following the vaccination process (9–12). Within intrinsic mechanisms, myeloid-derived suppressor cells (MDSCs) (13, 14) and CD4^+^FoxP3^+^ regulatory T cells (Tregs) (15) could be significant components of sexual dimorphism (16, 17). MDSCs are expanded under diverse conditions, such as sterile inflammation, cancer, infections, pregnancy, and after immunizations (18–20). Together with Tregs, they can influence the immunologic balance (18). However, the precise role of the intrinsic and extrinsic factors in sexual dimorphism in ChD and the efficacy of anti-*Tc* vaccines remain unclear.

Within this conceptual framework, our study aimed to investigate the influence of sex on the immunogenicity and protective efficacy of a promising mucosal vaccine based on the antigen *trans*-sialidase (TS) against *Tc* (19–21), with particular emphasis on both extrinsic and intrinsic regulatory mechanisms.

## Materials and methods

### Mice

BALB/c male and female mice (6–8 weeks old) were housed in High-Efficiency Particulate Air (HEPA)-ventilated racks under a 12:12-h light/dark cycle, with controlled temperature and humidity, and provided food and water *ad libitum*. All studies were approved by the Institutional Animal Care and Use Committee (Res. No. 0805/2020 and 2142/2024).

### Recombinant antigen, and adjuvant and immunization schedules

The N-terminal sequence of TS (GenBank: MZ215730.2) was obtained as described previously (19, 20). The adjuvant used was a cyclic-di-adenylate known as c-di-AMP (Air Fresh, Argentina, -A-, Sigma-Aldrich).

BALB/c mice (n = 4–9/group/sex) were immunized intranasally with three doses, 2 weeks apart, of the following formulations: a) saline [vehicle (V)], b) 10 µg of TS (TS), c) 10 µg of TS + 5 µg of c-di-AMP (TS+A), and d) 5 µg of c-di-AMP (A). Each dose (20 µL) was administered intranasally (10 µL/nostril) using a micropipette. Fifteen days after the final dose, mice were euthanized to assess humoral and cellular immunogenicity, and separate groups were infected for protection evaluation 28 days post-infection (pi).

### Determination of TS-specific antibodies

Fifteen days after the final dose, TS-specific antibodies were assayed in plasma, feces, and nasal lavages. ELISA microplates (Nunc-Inmuno Maxisorp™, Thermo) were coated with TS (0.5 µg) diluted in carbonate–bicarbonate buffer (0.05 M; pH 9.6) and incubated overnight. Anti-TS antibodies were detected using rat anti-mouse IgG_1_ and IgG_2a_-Horseradish Peroxidase (HRP) (1:2,000) or biotinylated rat anti-mouse IgA (1:1,500) mixed with streptavidin-HRP (1:1,500) (BD Biosciences). Absorbance was measured at 450/545 nm using an ELISA reader. Antibody levels are expressed as relative OD, with each OD value normalized by dividing it by the average OD of the V group.

### Delayed-type hypersensitivity test

Intradermal inoculation of 5 µg of TS was performed in the rear footpad 15 days after the final immunization. Hindpaw swelling was measured using a digital caliper before TS inoculation and at 24, 48, and 72 h post-inoculation, as previously described (19).

### Orchiectomy

Orchiectomy was performed 30 days before the first immunization. Male mice were anesthetized with ketamine (100 mg/kg)/xylazine (10 mg/kg) and randomly sham-operated (Ms) or subject to orchiectomy (Gx). Total testosterone levels were measured using an electrochemiluminescence immunoassay (ECLIA; Roche) to confirm the success of surgical castration. Only Gx animals with testosterone levels below 0.03 ng/mL were included in the study.

### Flow cytometry

To evaluate immunogenicity, splenocytes were stimulated with ionomycin and brefeldin-A (BD-GolgiPlug, BD Pharmingen) for 4 h and then specifically stimulated with TS for an additional 72 h (21). Then, splenocytes were blocked with anti-FcγII/III-R and stained with the following monoclonal antibodies: anti-CD4/PE-Cy7, anti-CD8/PerCP, anti-IFN-γ/FITC, anti-ROR-γt/PE, anti-Ki67/FITC, anti-CD44/APC-Cy7, and anti-CD62L/APC (BD Pharmingen). To determine both monocytic (M-MDSC: CD11b^+^/Ly6G^−^Ly6C^+^) and granulocytic (G-MDSC: CD11b^+^/Ly6G^+^Ly6C^+/low^) MDSCs after infection, splenocytes were stained with anti-CD11b/PerCP, anti-Ly6C/FITC, and anti-Ly6G/PE (BD Pharmingen). Tregs were determined with anti-CD4/PerCP and anti-FoxP3/PE (eBioscience). MDSC-resembling cells were also evaluated in blood. Plasma cytokine levels were assessed by Cytometric Bead Array (BD Biosciences). All samples were analyzed using a BD FACSAriaII flow cytometer.

### Oral infection and follow-up

Fifteen days after the last immunization, mice were deprived of water for 4 h and then orally challenged with 3,000 trypomastigotes (Tulahuen strain, TcVI). Parasitemia and clinical scores were evaluated as previously described until day 28 pi (19) (for further details, see **Supplementary Material S4a**). In two experimental rounds, half of the animals in each group were sacrificed on day 21 for histological and parasitological assessments.

### Tissue parasite burden

DNA was extracted from the heart, skeletal muscle, and small intestine following the method described by Cummings and Tarleton (22). PCR reactions were performed using HOT-FIREPol-EvaGreen qPCR MixPlus (Solis BioDyne) in StepOne™ Real-Time (Applied Biosystems), and for further details, see **Supplementary Material S4b**.

### Histopathology

The heart, skeletal muscle, and liver were collected 21 days pi, fixed in formalin, and embedded in paraffin. Five-micron sections were stained with hematoxylin and eosin to assess parasitism and inflammatory infiltration (for further details, see **Supplementary Material S4c**).

### Statistical analyses

Data were analyzed using non-parametric tests (Kruskal–Wallis followed by Mann–Whitney U-test) using the GraphPad-Instat 4.0 software. Data are representative of at least two independent experiments (n = 3–9 mice/group). Differences between groups were considered significant when the p-value was <0.05.

## Results

### Sex-based differences in the immunogenicity of the TS+A vaccine

#### Systemic and mucosal TS-specific humoral response

**Figure 1:**
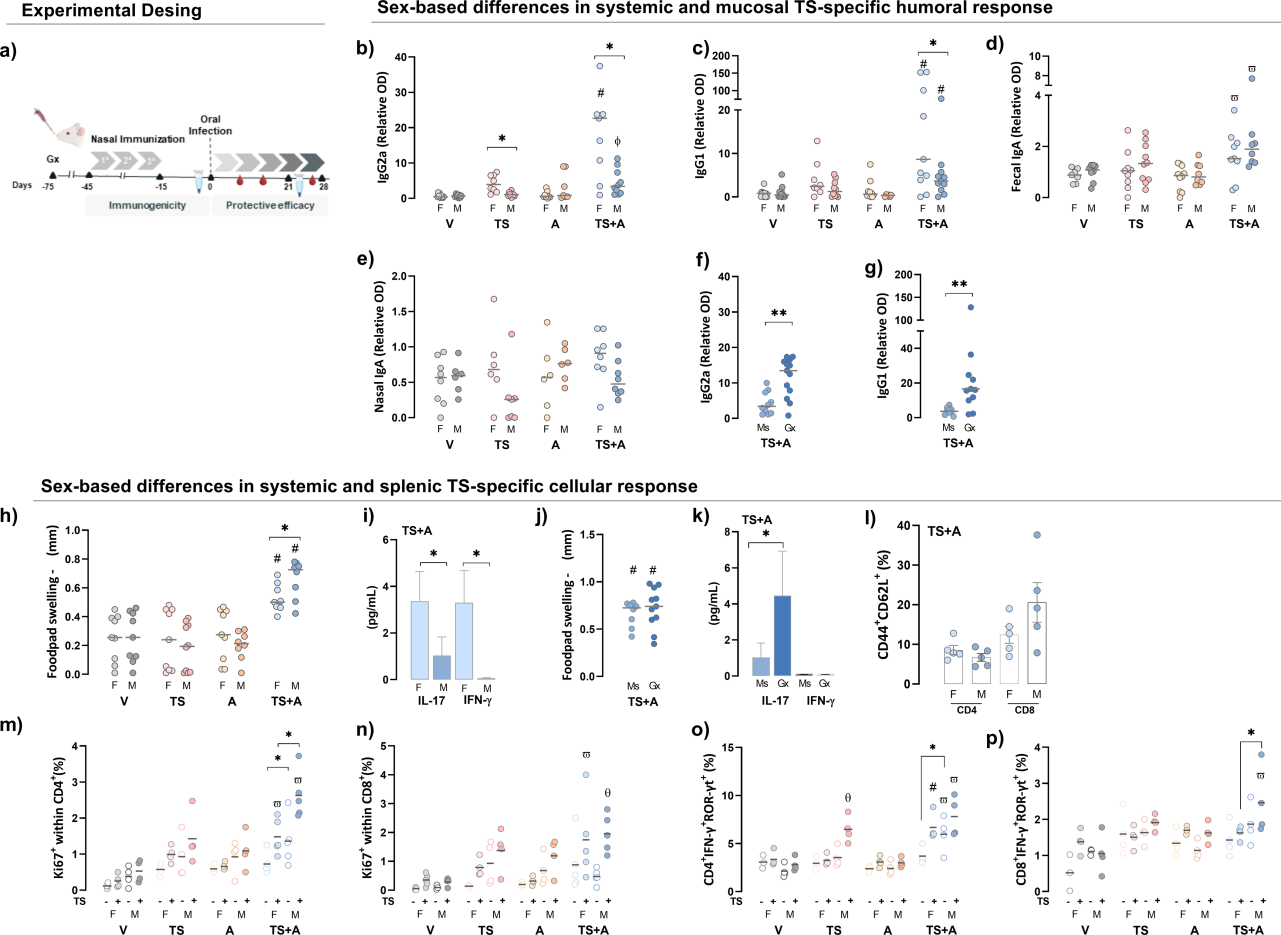
Sex-based differences in systemic and mucosal immunogenicity after vaccination. Female (F) and male (M) BALB/c mice were immunized with vehicle (V), *trans*-sialidase (TS), c-di-AMP (A), or TS combined with A (TS+A). Immunogenicity was assessed 15 days after completing the immunization schedule. Experimental design scheme **(a)**. Humoral immunogenicity: TS-specific IgG_2a_ **(b)** and IgG_1_ **(c)** were evaluated in plasma. TS-specific IgA was evaluated in fecal **(d)** and nasal lavages **(e)**. Plasma IgG_2a_ **(f)** and IgG_1_ **(g)** levels were also analyzed in gonadectomized (Gx) and sham-operated TS+A-vaccinated mice (Ms). Cellular immunogenicity: footpad swelling after 72 h of TS intradermal challenge (Δ in mm) among vaccinated groups **(h)**. IL-17 and IFN-γ plasma levels in the TS+A groups **(i)**. Footpad swelling after 72 h of TS challenge (Δ in mm) in TS+A-vaccinated Ms and Gx mice **(j)**. IL-17 and IFN-γ plasma levels in the TS+A Ms and Gx groups **(k)**. Central memory T cells (T_CM_, CD44^+^CD62L^+^) following *in vitro* TS antigen stimulation of splenocytes from TS+A animals. Data represent the net increase in T_CM_ frequency after subtraction of baseline levels from the corresponding unstimulated controls **(l)**. Proportion of splenic Ki67^+^CD4^+^ **(m)**, Ki67^+^CD8^+^ **(n)**, CD4^+^IFN-γ^+^ROR-γt^+^ **(o)**, and CD8^+^IFN-γ^+^ROR-γt^+^ T cells **(p)** after *ex vivo* re-stimulation with TS (solid circles), with open circles representing the corresponding unstimulated control. Data are expressed as mean ± SEM. Statistical significance: *p < 0.05 between sexes; ^#^p < 0.05 TS+A compared to the rest of similar sex groups; ^θ^p < 0.05 TS versus V; ^ф^p < 0.05 TS+A versus TS and V; ^ω^p < 0.05 TS+A versus A and V.

Following immunization, TS+A-vaccinated female mice exhibited the highest levels of circulating TS-specific IgG_2a_ and IgG_1_ among all groups, whereas in males, this was observed only for IgG_1_. In addition, females consistently showed higher levels than males (**Figures 1b, c**). As a surrogate marker of the mucosal humoral response, IgA was measured in fecal and nasal lavage samples. TS-specific fecal IgA was elevated in the TS+A group compared to the V and A groups, regardless of sex (**Figure 1d**). No differences in TS-specific IgA were observed in nasal lavages (**Figure 1e**). In TS+A-vaccinated males, testosterone negatively influenced TS-specific IgG_2a_ and IgG_1_ levels (**Figures 1f, g**) but had no effect on IgA (**Supplementary Material S1a, b, S3**).

#### Systemic and splenic TS-specific cellular response

The TS-specific delayed-type hypersensitivity (DTH) response was stronger in TS+A-vaccinated males than females at 72 h, with no differences in the V, TS, and A groups regardless of sex (**Figure 1h**). Concurrently, TS+A-vaccinated females had higher plasma IL-17 and IFN-γ levels than males (**Figure 1i**). Castration did not affect the DTH response in TS+A-vaccinated males (**Figure 1j**) but increased circulating IL-17 levels in the same group (**Figure 1k**). In TS+A-vaccinated mice, TNF-α, IL-6, IL-4, and IL-2 levels remained unchanged between the sexes and unaffected by testosterone depletion in males (**Supplementary Material S1c, d, S3**). TS+A-vaccinated mice showed an increase in both CD4^+^ and CD8^+^ central memory T cells (T_CM_), with a slight tendency toward higher TS-specific CD8^+^ T_CM_ frequency in males compared to females (**Figure 1l**). In addition, the TS-driven proliferative response was also evidenced by an increase in both Ki67^+^CD4^+^ and Ki67^+^CD8^+^ T cells (**Figures 1m, n**). However, the effect was more pronounced in CD4^+^ T cells from TS+A-vaccinated males than females (**Figure 1m**), while specific CD8^+^ T-cell proliferation remained comparable between sexes (**Figure 1n**). Similarly, after *ex vivo* TS re-stimulation, an enrichment of CD4^+^IFN-γ^+^ROR-γt^+^ and CD8^+^IFN-γ^+^ROR-γt^+^ T cells, where ROR-γt serves as a surrogate marker for IL-17, was observed in both sexes, with a tendency toward higher levels in males (**Figure 1o, p**). Notably, the proliferation of splenic CD4^+^ and CD8^+^ T cells from Gx-TS+A-vaccinated males was lower compared to that of Ms-TS+A males after *ex vivo* TS re-stimulation (**Supplementary Material S1e, f, S3**).

### Sex-based differences in protective efficacy of the TS+A vaccine in orally infected mice

#### Parasitemia and clinical score

**Figure 2:**
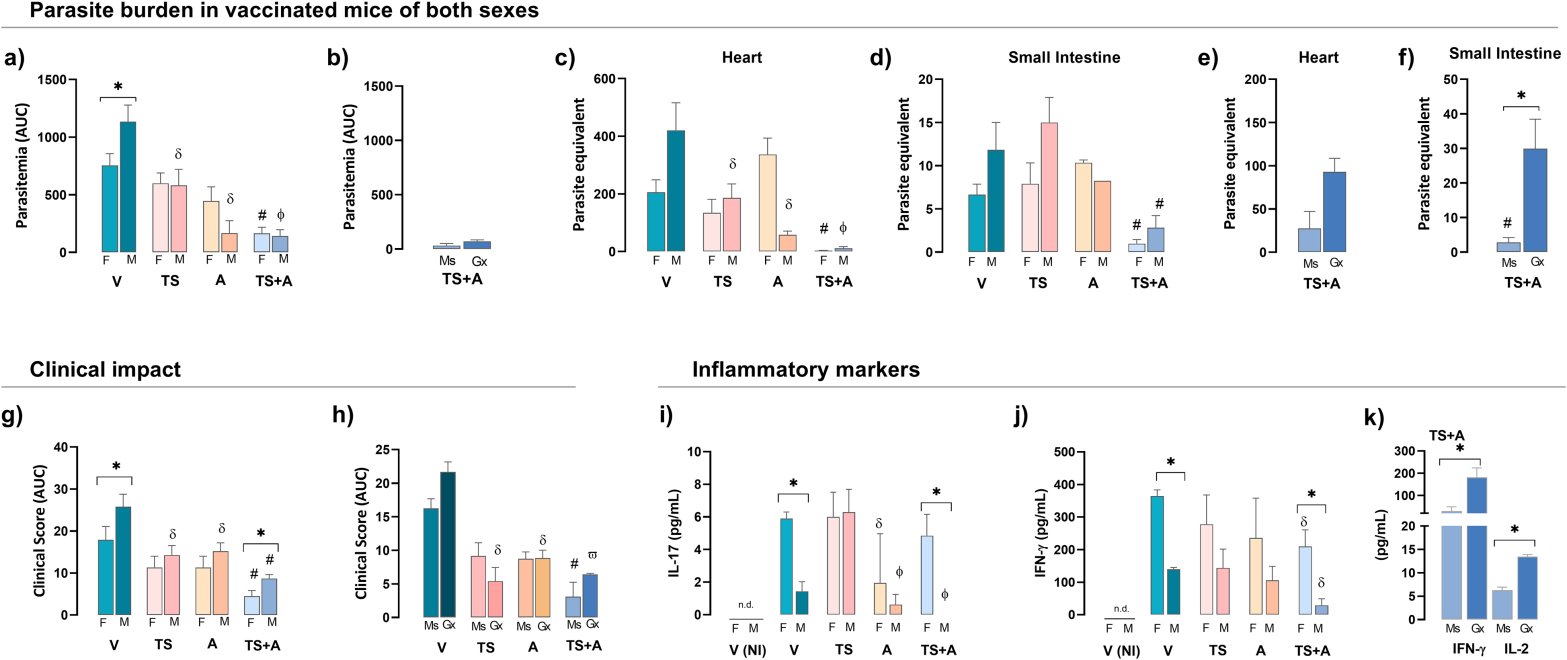
Protective efficacy of vaccination in both sexes. Female (F) and male (M) BALB/c mice vaccinated with vehicle (V), *trans*-sialidase (TS), c-di-AMP (A), or TS combined with A (TS+A) were orally infected with 3,000 trypomastigotes 15 days later. Parasite burden: cumulative parasitemia, expressed as the area under the curve (AUC), was assessed in F and M **(a)** and TS+A gonadectomized (Gx) compared to sham-operated mice (Ms) **(b)**. Heart **(c)** and small intestine **(d)** parasite loads were analyzed in both sexes and TS+A Ms and Gx mice (e, f). Clinical impact: clinical signs were scored daily, as follows: no signs (#0), piloerection (#1), hunchback (#1.5), eye discharge (#2), decreased activity (#2.5), and diarrhea (#3). Individual daily scores were summed to determine the total clinical score for each animal. The cumulative clinical score (AUC) was calculated for each group and both sexes **(g)** and Ms and Gx mice **(h)**. Plasma levels of IL-17 **(i)** and IFN-γ **(j)** were measured in F and M and in TS+A Gx and Ms mice **(k)**. Data are expressed as mean ± SEM. Statistical significance: *p < 0.05 between sexes; ^#^p < 0.05 TS+A compared to the rest of similar sex groups; ^δ^p < 0.05 versus V, ^ф^p < 0.05 TS+A versus TS or V; ^ω^p < 0.05 TS+A or TS versus A and V; n.d. not detected.

Consistent with previous reports, BALB/c female mice exhibited greater resistance to *Tc* infection than males (4), even after oral infection, as shown here. Indeed, unvaccinated and orally infected V females displayed twofold lower parasitemia levels compared to infected V males (**Figure 2a**). In both sexes, TS+A vaccination resulted in a significant reduction in parasitemia compared to that in the V group, while TS or A vaccination alone provided an intermediate level of control (**Figure 2a**). In TS+A males, castration did not affect parasitemia control (**Figure 2b**). Additionally, TS+A vaccination reduced parasite load in the heart (**Figure 2c**), small intestine (**Figure 2d**), and skeletal muscle (**Supplementary Material S2a, S3**) in both sexes. However, in TS+A-infected males, testosterone depletion resulted in a similar, although non-significant, trend in parasite load in the heart (**Figure 2e**) and skeletal muscle (**Supplementary Material S2b, S3**) but caused a marked increase in the intestine (**Figure 2f**).

Infected V mice exhibited clear signs of clinical outcome, although less pronounced in females compared to males (**Figure 2g**). Despite this, TS+A vaccination provided strong protection against the development of clinical outcomes in both sexes, with the protective effects being more pronounced in females (**Figure 2g**). Regardless of sex, the TS- and A-infected groups showed an intermediate level of clinical outcome compared to the V- and TS+A-infected groups (overall, p < 0.05) (**Figure 2g**). Castration did not influence clinical scores in TS+A males (**Figure 2h**).

Particularly in the V- and TS+A-infected groups, females exhibited higher systemic levels of IFN-γ and IL-17 than males (**Figures 2i, j**). In TS+A-infected males, the absence of testosterone enhanced circulating IFN-γ along with a slight increase in IL-2 (**Figure 2k**). In TS+A-vaccinated animals, the plasma levels of TNF-α, IL-6, IL-4, and IL-2 showed no differences between sexes, nor between Gx and Ms males (**Supplementary Material S2c, d, S3**).

#### Sex-based modulation of MDSCs and Tregs by the TS+A vaccine

**Figure 3:**
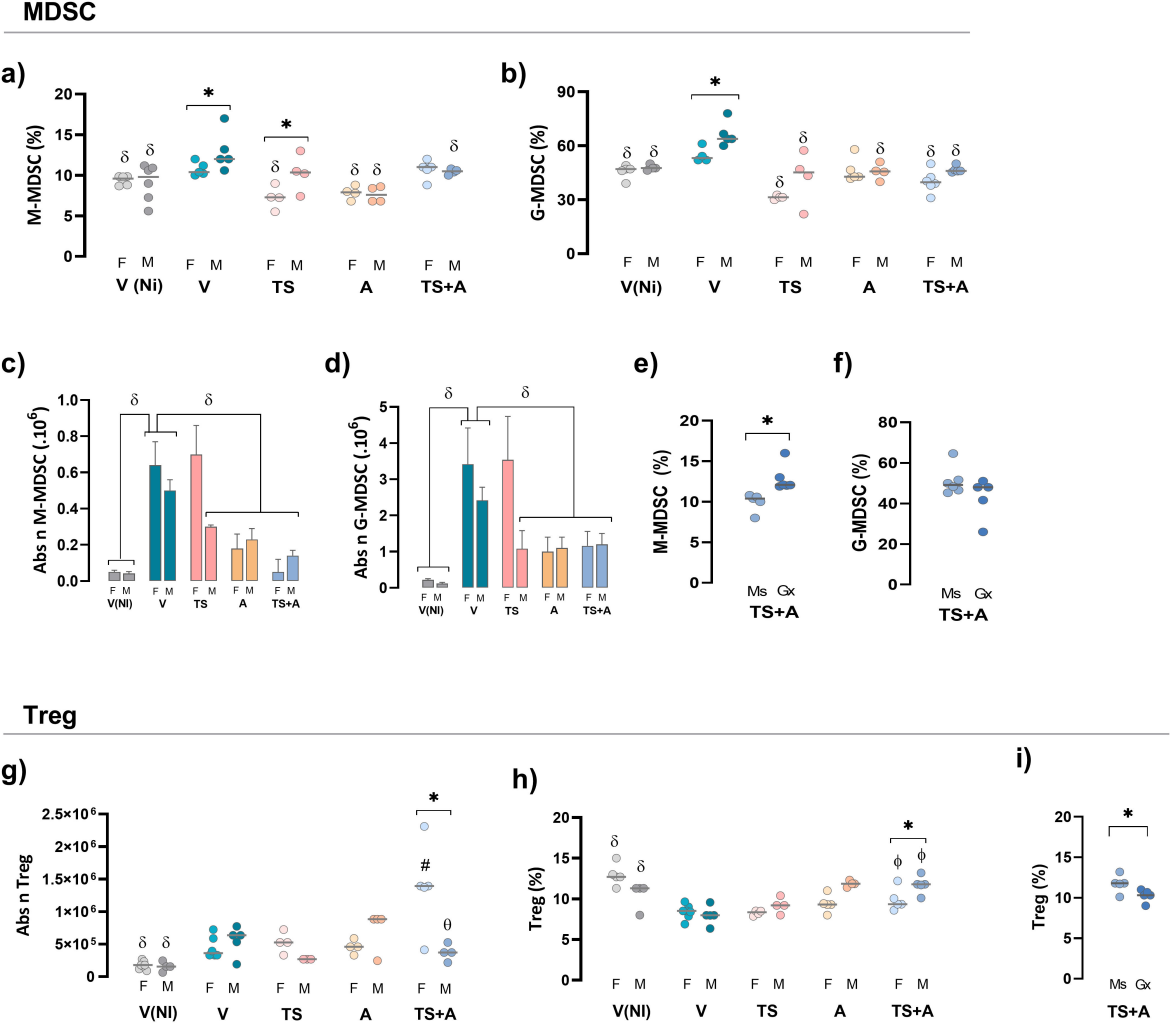
Regulatory response in vaccinated mice of both sexes challenged with *Trypanosoma cruzi* (*Tc*). After 21 days post-infection, the frequency and absolute number of regulatory populations were evaluated. Myeloid-derived suppressor cells (MDSCs): splenic monocytic (M)-MDSC and granulocytic (G)-MDSC cell frequencies **(a, b)** and absolute numbers **(c, d)**. Frequencies of MDSC in TS+A gonadectomized (Gx) and sham-operated (Ms) mice **(e, f)**. Regulatory T cells (Treg cells): absolute numbers **(g)** and frequencies **(h)** of splenic CD4^+^FoxP3^+^ Treg cells were determined for both sexes. Frequency of Treg cells was recorded in TS+A Gx and Ms mice **(i)**. Data are expressed as mean ± SEM. Statistical significance: *p < 0.05 between sexes; ^#^p < 0.05 compared to the rest of similar sex groups; ^δ^p < 0.05 versus V; ^ф^p < 0.05 TS+A versus TS and V.

To evaluate the impact of sex on the intrinsic regulatory response in vaccinated mice, MDSCs and Tregs were analyzed at day 21 pi. A control V non-infected group, V(NI), was added for comparative purposes. As expected, both the proportions and absolute numbers of splenic M-MDSC and G-MDSC populations increased following infection, with higher levels observed in V-infected mice compared to the V(NI) controls (**Figures 3a–d**, gating strategy in **Supplementary Material S2e, S3**). The increase in frequency in both MDSC populations was more pronounced in V males than in V females (**Figures 3a, b**). Strikingly, vaccination with all formulations prevented the infection-induced increase in splenic MDSC frequency in both sexes (**Figures 3a, b**). In addition, both TS+A-vaccinated females and males maintained M-MDSC absolute numbers comparable to those of the V(NI) groups, with a similar trend observed in G-MDSCs (**Figures 3c, d**). Despite that, castration negatively affected the effects conferred by TS+A vaccination upon splenic M-MDSCs, but not G-MDSCs (**Figures 3e, f**). The evaluation of blood-resembling MDSCs showed a slight increase in the frequency of G-MDSCs in V mice, which was not counteracted by TS+A vaccination, but no change was observed in M-MDSCs (**Supplementary Material S2f, g, S3**).

In both sexes, oral infection in the V groups resulted in a slight increase in the absolute numbers of splenic Tregs but significantly reduced their frequency when compared with the V(NI) groups (**Figures 3g, h**; gating strategy in **Supplementary Material S2h, S3**). This finding aligns with results from studies where infection was induced through routes other than oral (22, 23). In both sexes, TS+A vaccination increased the absolute numbers of splenic Tregs compared to V(NI) mice. This effect was also more pronounced in TS+A-vaccinated females than in V females (**Figure 3g**). Notably, in both sexes, TS+A vaccination prevented the decline in Treg frequency caused by oral infection, whereas TS or A alone only partially mitigated this effect (**Figures 3g, h**). However, when comparing TS+A-vaccinated females and males, this effect was slightly more pronounced in males (**Figure 3h**) but was avoided when testosterone was previously depleted (**Figure 3i**).

#### Mitigation of sex-based differences in histological damage by the TS+A nasal vaccine

**Figure 4:**
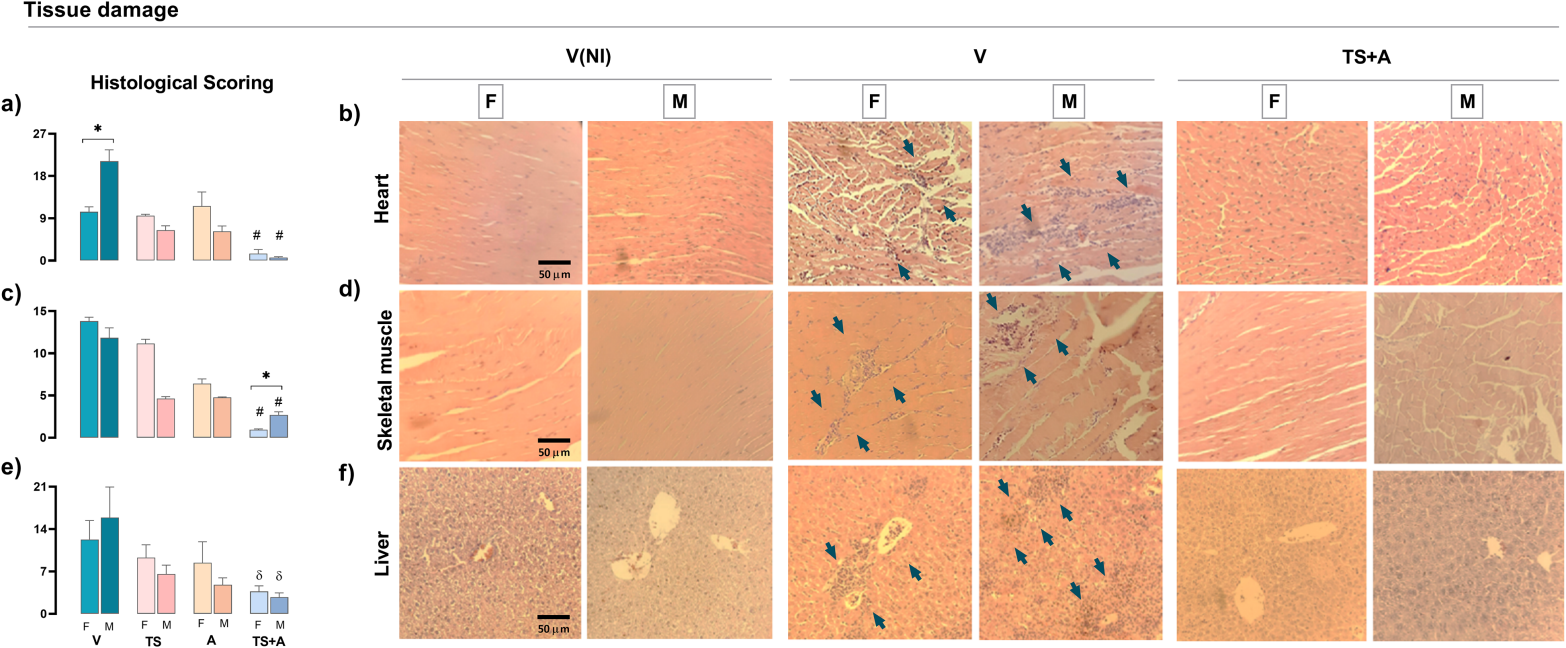
Protective effects of TS+A vaccination upon tissue damage. Histological damage was evaluated from hematoxylin and eosin-stained organ sections from heart, skeletal muscle, and liver after 21 days post-infection. Global histological scores for each tissue were calculated as the proportion of inflammatory infiltrate (infiltrated area/total area) from 10 microscopic images at ×20 magnification, multiplied by the severity score assigned to the infiltrate (1 = mild, 2 = moderate, and 3 = severe). Heart global score from vaccinated and orally infected (F) female and (M) male mice **(a)**. Heart representative images from V(NI), V, and TS+A mice of both sexes **(b)**. Similar evaluations were made for skeletal muscle **(c, d)** and liver (e, f). Arrows indicate inflammatory infiltrates or areas of tissue destruction. Data are expressed as mean ± SEM. Statistical significance: *p < 0.05 between sexes; ^#^p < 0.05 compared to the rest; ^δ^p < 0.05 versus V.

Histopathological analysis of the hearts in the V-infected groups revealed more pronounced damage in males compared to females, as indicated by the severity of inflammation and the extent of inflammatory infiltration (**Figures 4a, b**). Interestingly, these sex differences were no longer observed in the TS+A-vaccinated and orally infected animals, which showed significantly reduced myocarditis, with comparable scoring in both sexes (**Figures 4a, b**). Likewise, TS+A vaccination effectively minimized tissue damage in the skeletal muscle (**Figures 4c, d**) and liver, regardless of sex (**Figures 4e, f**). Notably, in Gx-TS+A males, myocarditis remained at low levels despite the absence of testosterone (**Supplementary Material S2i, S3**).

## Discussion

Diverse baseline sex differences in immune responses have been reported (24, 25). However, the contribution of sex-driven differences to vaccine efficacy and effectiveness has only recently gained attention. In this regard, some studies have shown that after vaccination, females exhibited stronger immunoreactivity, while males tended to mount less immunogenic responses (7, 11, 26, 27). Therefore, there is a growing consensus that ongoing research should take into account the role of sex in shaping vaccine outcomes to enhance vaccine development (28). Despite this, no studies have yet addressed sex differences in experimental vaccine responses against ChD. To fill this gap, we examined how sex influences immunogenicity and protective responses in BALB/c mice following the nasal administration of a TS-based vaccine. Additionally, given the well-known immunosuppressive capacity of testosterone, we specifically evaluated its role in the immune response triggered by the TS+A vaccine.

Regarding humoral immunogenicity, our findings showed that TS+A administration elicited a stronger TS-specific response in females than in males. This aligns with evidence from human and animal models demonstrating that females generally produce higher antibody levels after vaccination (29–33). Additionally, the elevated TS-specific plasma IgG_2a_ levels observed in TS+A-immunized females appear to be linked to the vaccine’s ability to enhance systemic IFN-γ and IL-17 levels compared to those in males. Both cytokines were involved in promoting isotype class switching to IgG_2a_ and may also contribute to IgG_1_ production (34). Likely, in TS+A-vaccinated female mice, estradiol may play a key role in driving the robust TS-specific IgG_2a_ and IgG_1_ responses, as this hormone promotes class-switch recombination and high-affinity antibody production (29). However, despite reports suggesting a potential sex effect on IgA secretion (35), we did not observe any sex differences in the mucosal secretion of TS-specific IgA, indicating that sex-related vaccine effects on humoral responses are more pronounced in plasma. In line with this, studies have documented sex disparities in systemic and mucosal responses to vaccine antigens in preclinical models of viral infections, including genital HSV-2, HIV/SIV, and COVID-19 (36, 37).

Conversely, our findings highlight the inhibitory role of testosterone in humoral responses, particularly regarding TS-specific plasma antibodies in TS+A-vaccinated males. Testosterone depletion in this group enhanced IgG_2a_- and IgG_1_-specific responses, with TS-specific IgG_2a_ levels in castrated TS+A-vaccinated mice reaching levels comparable to those in TS+A-vaccinated females. Furthermore, this enhancement in castrated TS+A-vaccinated mice was associated with increased IL-17 levels. Testosterone is known to inhibit B-cell activating factor (BAFF)-mediated antibody production (38), antagonize NF-κB and AP-1 functions (39), reduce IFN-γ, and suppress IL-17 (40, 41). These processes collectively may explain the reduced TS-specific IgG_2a_ and IgG_1_ production in TS+A-vaccinated males compared to females.

Interestingly, despite its suppressive effects on antibody production, testosterone appears to enhance cell-mediated immune responses following TS+A vaccination. A male bias in this response was observed, as evidenced by more pronounced and sustained footpad swelling after the TS challenge. Additionally, since androgens can promote Th1 response (42), this could explain the higher splenic TS-specific CD8 T_CM_ and CD8^+^IFN−γ^+^ROR−γt^+^ proportions and the slightly enhanced T_CM_ response in the TS+A males. Furthermore, the reduced proliferative response of CD4+ T cells in castrated TS+A-vaccinated mice supports the idea that testosterone plays a role in modulating this response. However, one limitation of this study is the lack of data on the vaccine’s effects on long-term memory in both sexes. Therefore, future studies evaluating immunogenicity and long-term protection should also consider evaluating the T- and B-cell memory subgroups.

Regarding the effects of sex on the development of human ChD, available reports are limited. However, existing evidence suggests that progression to cardiomyopathy is more common in men (43, 44). Most of the evidence on sex-based differences in susceptibility to *Tc* infection comes from animal models mimicking vector transmission, where males generally exhibit greater susceptibility, as evidenced by higher parasitemia levels and shorter survival times compared to females (4, 6, 45). Notably, oral transmission involved a different entry route and likely distinct immunological mechanisms, and to the best of our knowledge, there are no available data on whether sexual dimorphism is preserved in this context. Given that unvaccinated V males exhibited higher parasitemia, a more severe acute infection course, and more pronounced cardiac damage compared to unvaccinated V females, we confirm that sex-related differences in susceptibility persist even in the context of experimental oral transmission.

In the present study, the host’s sex emerged as a critical factor influencing the immunogenicity of the TS+A vaccine. However, these sex-related differences were surpassed, as the TS+A vaccine provided robust and comparable protection in both sexes, significantly reducing parasite load and tissue damage, and ultimately resulting in more favorable outcomes for both males and females. Notably, the significant decrease in parasite burden observed in TS+A-vaccinated mice appears to contribute to the mitigation of experimental chronic chagasic myocarditis progression in both sexes, as evidenced by diminished inflammatory infiltration in the myocardium (46–49). In contrast, female mice exhibited higher IL−17 levels than males in both the V and TS+A groups, consistent with estrogen−driven enhancement of Th17 responses. Interestingly, previous studies conducted during the chronic stage of infection in TS+A-vaccinated females (21) showed that the TS+A vaccine does not sustain elevated IL-17 levels at this stage. Since IL-17 can drive inflammation in persistent infections (50), their diminution together with that of other inflammatory cytokines may contribute to reduced chronic tissue damage (51, 52).

However, immune evaluations following infection, while consistent with immunogenicity findings, do not fully explain the similarly reduced levels of parasitism and heart cell infiltration observed in TS+A-vaccinated females and males. These findings may, in part, be explained by the TS+A vaccine’s ability to sustain the frequency of TS-specific Tregs in levels comparable to those of uninfected animals in both sexes, thereby helping to control tissue damage. These findings align with previous studies indicating that in *Tc*-infected mice, an excessive T-effector response can overwhelm the amounts and functions of Tregs, increasing myocarditis and lethality (53, 54). TS-based vaccines, however, could help balance the Teff/Treg ratio, enhancing the immune system’s capacity to combat the parasite while preventing tissue damage (55, 56).

Previous studies have demonstrated that a more severe course of *Tc* infection has been linked to MDSC enhancement (46, 57, 58). Furthermore, the increase in MDSC populations seems to negatively affect vaccine efficacy against *Tc* (48, 49). In this study, we observed that, regardless of sex, oral *Tc* infection in unvaccinated mice led to a significant increase in both subsets of splenic MDSCs, with this effect being more pronounced in males. Consistent with this, there are reports associating testosterone with the induction of MDSCs (59). Interestingly, the TS+A vaccine appears to primarily prevent the expansion of M-MDSCs, which could facilitate effective parasite clearance, thereby reinforcing the idea that controlling MDSC levels is crucial for the protective efficacy of vaccination. This effect may arise from the vaccine’s ability to counteract the MDSC-driven suppression of TS-specific T-cell priming and activation. Alternatively, by preventing the increase in MDSCs, the TS+A formulation may also promote the expansion of Tregs, ultimately contributing to significant protection in both immunized and infected mice (48, 49, 55, 56).

Here, we demonstrated that testosterone influences TS+A immunogenicity, contributing to the observed sex differences in antibody production, T-cell activation, inflammatory cytokine levels, and M-MDSCs. These findings are supported by systems-wide approaches to vaccine efficacy, which have identified testosterone as a key modulator of the reduced pathogen-neutralizing activity observed in males (60).

Another limitation of this study is its focus on testosterone depletion as a strategy to enhance vaccine immunogenicity in males, without considering the role of estradiol in females. Future studies evaluating the effects of estradiol, through either depletion or supplementation, on the TS+A vaccine response could offer a more comprehensive understanding of how sex hormones influence vaccine-induced immunity.

Overall, our findings highlight sex-based differences in immunogenicity following TS+A immunization, as well as the presence of sexual dimorphism in the natural course of oral *Tc* infection. However, despite these differences, our results suggest that prophylactic TS+A immunization is beneficial for both sexes, improving the clinical course of acute infection by reducing parasite load and tissue damage. Ultimately, this results in comparable outcomes in both males and females, underscoring the effectiveness of TS+A vaccination irrespective of sex.

## Data Availability

The raw data supporting the conclusions of this article will be made available by the authors, without undue reservation.
