# Supplementary figures and images for: Sexual dimorphism-driven differences are overcome in a preclinical vaccine model against Trypanosoma cruzi
Source: Front Immunol. 2025 Jun 26;16:1526573. doi: 10.3389/fimmu.2025.1526573 (PMC12241810; doi:10.3389/fimmu.2025.1526573)

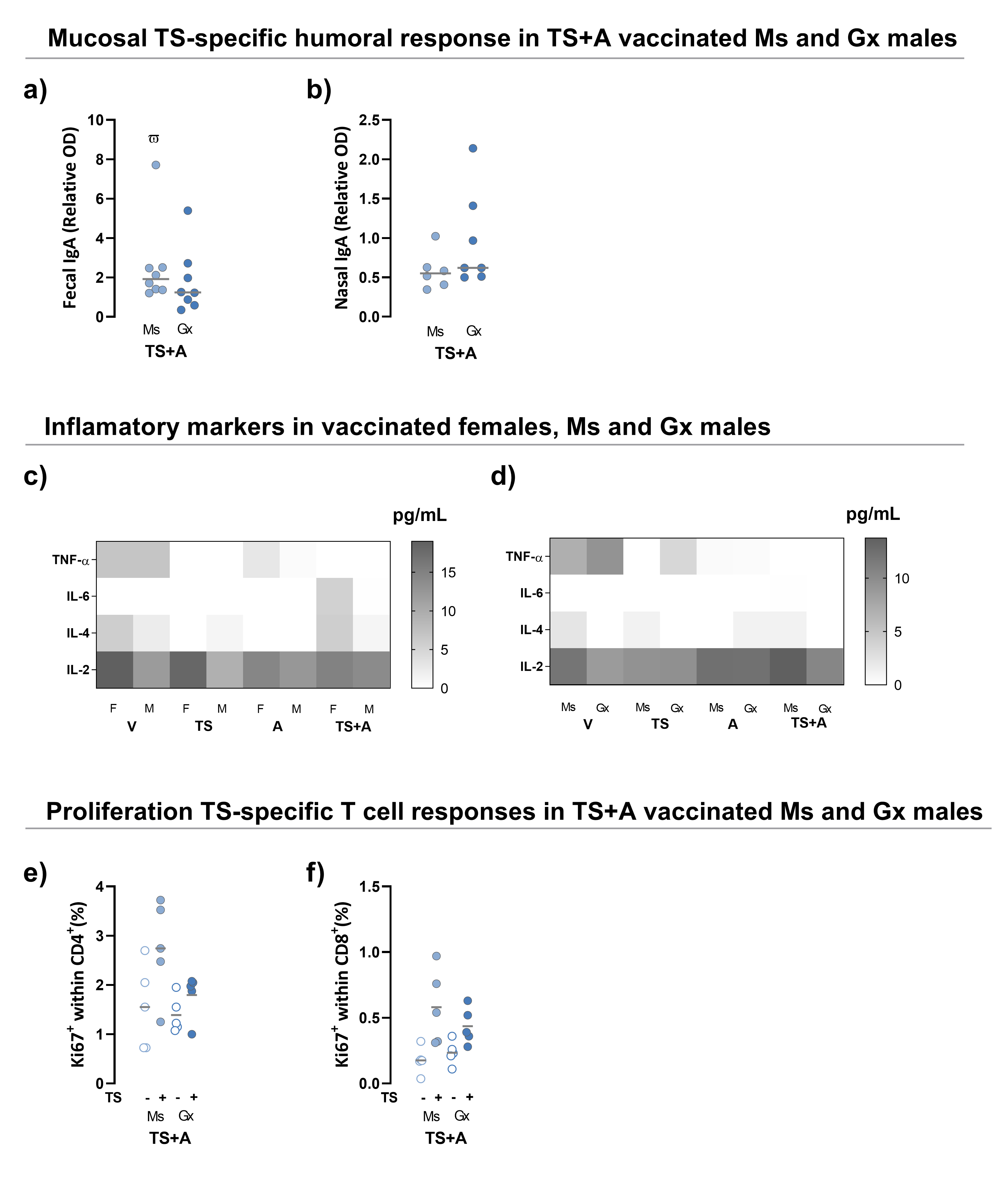

Supplement: Supplementary file 1 [file SupplementaryFile1.tif]
