# Supplementary material for: Sexual dimorphism-driven differences are overcome in a preclinical vaccine model against Trypanosoma cruzi
Source: Front Immunol. 2025 Jun 26;16:1526573. doi: 10.3389/fimmu.2025.1526573 (PMC12241810; doi:10.3389/fimmu.2025.1526573)
Supplement: Supplementary file 3 [file SupplementaryFile3.docx]

**Legends of Supplementary Figures**

**Figure S1:** **TS-specific immunogenicity.** TS-specific IgA levels were measured in fecal **(a)** and nasal lavages **(b)** from TS+A gonadectomized -Gx- and sham-operated -Ms- males. Plasma cytokine levels were measured using a cytometric bead array and presented as a heatmap, with concentrations represented from white (low) to dark gray (high). Comparisons were made between female -F- and male -M- mice **(c)**, as well as between Gx and Ms males **(d)**. *P*roliferation of splenic CD4+ **(e)** and CD8+ T-cells from TS+A Gx and Ms males **(f)** after *ex vivo* re-stimulation with TS. Statistical significances: *p<0.05 between sexes; ^#^p<0.05 compared to the rest; ^ፀ^p<0.05 versus V; ^ф^p<0.05 TS+A versus TS and V; ^ω^p<0.05 TS+A or TS versus A and V. Vehicle (V), Trans-sialidase (TS), c-di-AMP (A), TS combined with A (TS+A).

**Figure S2: Protective efficacy.** Mice were immunized with vehicle (V), trans-sialidase (TS), c-di-AMP (A), or TS combined with A (TS+A) and, 15 days later, were orally infected with 3000 trypomastigotes. Parasite burden in skeletal muscle: comparisons were made between female -F- and male -M- mice **(a)**, as well as between gonadectomized -Gx- and sham-operated -Ms- males **(b)**. Inflammatory markers: Plasma cytokine levels were assessed using a cytometric bead array, represented as heatmaps showing concentrations ranging from white (low) to dark gray (high) for F and M **(c)** and for Gx and Ms males **(d).** Gating strategy for identifying myeloid-derived suppressor cells (MDSCs) **(e)**. Blood-resembling monocytic and granulocytic MDSC frequency in F and M (f, g). Gating strategy for splenic CD4+FoxP3+ regulatory T-cells (Treg) **(h)**. Heart global score from vaccinated and orally infected Gx and Ms males **(i)**.
