# Supplementary material for: Sexual dimorphism-driven differences are overcome in a preclinical vaccine model against Trypanosoma cruzi
Source: Front Immunol. 2025 Jun 26;16:1526573. doi: 10.3389/fimmu.2025.1526573 (PMC12241810; doi:10.3389/fimmu.2025.1526573)
Supplement: Supplementary file 4 [file SupplementaryFile4.docx]

**S.4 Material and Methods**

**S.4a Oral infection and follow-up**

Fifteen days after the last immunization, mice were kept without water for 4h and then orally challenged with 3000 trypomastigotes (Tulahuen-TcVI) into the mouth following the methodology proposed by Barreto-de-Albuquerque et al. 2015^(25)^. For this challenge phase, the vehicle group (V) was split into two subgroups: infected (V) and non‑infected (V(NI)). Parasitemias were evaluated as described earlier^(21)^. To assess clinical manifestations along infection, a clinical scoring system was employed(21). Briefly, the individual score is due to the sum of the pre-established values for each clinical manifestation, as follows: absence of signs:(#0), piloerection:(#1), hunchback:(#1.5), ocular involvement:(#2), decreased locomotor activity:(#2.5) and diarrhea:(#3). The global score was determined as the average of individual scores. The cumulative score for each group was represented as the area under the curve (AUC) over the experimental period.

**S.4b Tissue parasite burden**

DNA was extracted from the heart, skeletal muscle, and small intestine following the method described by Cummings and Tarleton^(24)^. DNA samples were adjusted to a final concentration of 25ng/μL and used as templates for qPCR reactions with specific primers targeting the kDNA: S36:(5′-GGTTCGATTGGGGTTGGTG-3′) and S67:(5′-GAACCCCCCTCCCAAAACC-3′). To generate the Tc quantification curve, 25mg of uninfected tissues were spiked with 1×10⁶ trypomastigotes. Total DNA was extracted, and the final concentration was adjusted to 25ng/μL. Standard curves were prepared through tenfold serial dilutions, using DNA from uninfected tissues (25ng/μL) as the diluent to generate a range from 0.01 to 1000 parasite equivalents per 50ng of total DNA. Parasite load was expressed as equivalent parasites/50ng of murine DNA. PCR reactions were performed using HOT-FIREPol-EvaGreen qPCR MixPlus (Solis-Biodyne) in StepOneTM Real-Time (Applied-Biosystems).

**S.4c Histopathology**

Hearts, skeletal muscle, and liver were collected 21 days pi, fixed in formalin, and paraffin embedded. Five-micron sections were stained with hematoxylin/eosin to assess parasitism and inflammatory infiltrates. The mean intensity of inflammation for each group was determined as the average of individual scores, scored as mild (#1), moderate (#2), or severe (#3)(21,25), by an experienced pathologist. The extension of inflammation was quantified by calculating the infiltrated area relative to total tissue area from 10 images at 20× magnification using ImageJ software. The global histological score was calculated by multiplying the infiltrated area proportion by the mean intensity of inflammation.
